# Time-Course Small RNA Profiling Reveals Rice miRNAs and Their Target Genes in Response to Rice Stripe Virus Infection

**DOI:** 10.1371/journal.pone.0162319

**Published:** 2016-09-14

**Authors:** Sen Lian, Won Kyong Cho, Sang-Min Kim, Hoseong Choi, Kook-Hyung Kim

**Affiliations:** 1 College of Crop Protection and Agronomy, Qingdao Agricultural University, Qingdao, Shandong, 266109, China; 2 Department of Agricultural Biotechnology, Research Institute of Agriculture and Life Sciences, and Plant Genomics and Breeding Institute, College of Agriculture and Life Sciences, Seoul National University, Seoul, 08826, Republic of Korea; 3 Crop Foundation Research Division, National Institute of Crop Science, Rural Development Administration, Wanju, 55365, Republic of Korea; National University of Singapore, SINGAPORE

## Abstract

It has been known that many microRNAs (miRNAs) are involved in the regulation for the plant development and defense mechanism by regulating the expression of the target gene. Several previous studies has demonstrated functional roles of miRNAs in antiviral defense mechanisms. In this study, we employed high-throughput sequencing technology to identify rice miRNAs upon rice stripe virus (RSV) infection at three different time points. Six libraries from mock and RSV-infected samples were subjected for small RNA sequencing. Bioinformatic analyses revealed 374 known miRNAs and 19 novel miRNAs. Expression of most identified miRNAs was not dramatically changed at 3 days post infection (dpi) and 7 dpi by RSV infection. However, many numbers of miRNAs were up-regulated in mock and RSV-infected samples at 15 dpi by RSV infection. Moreover, expression profiles of identified miRNAs revealed that only few numbers of miRNAs were strongly regulated by RSV infection. In addition, 15 resistance genes were targets of six miRNAs suggesting that those identified miRNAs and 15 NBS-LRR resistance genes might be involved in RSV infection. Taken together, our results provide novel insight into the dynamic expression profiles of rice miRNAs upon RSV infection and clues for the understanding of the regulatory roles of miRNAs via time-course.

## Introduction

Rice is one of economically important crops in the world and provides main food for more than half the world’s population. In addition, rice is a model research plant for monocotyledonous plants. The rice genome, which has relatively small genome of known cereal crops, has been sequenced. The size of rice genome is about 450 megabases containing more than 50,000 genes [1, 2].

*Rice stripe virus* (RSV) belongs to the genus *Tenuivirus* but has not yet been assigned to any virus family. RSV causes epidemic stripe disease on rice and results in serious damages on rice production in many Asian countries including China, Japan, and South Korea [3–5]. RSV is transmitted in a persistent and circulative-propagative manner by the insect vector small brown planthopper (SBPH) [3]. A recent demonstrated that viruliferous rate of SBPH is highly correlated with rice stripe disease epidemics [6]. In addition, the percentage of viruliferous infects was also seasonally changed [7]. The RSV genome consists of four single-stranded RNAs referred as RNA1 to RNA4 and encodes a total of seven proteins [8–11].

The microRNAs (miRNAs) are small non-coding RNA molecules with 19–24 nucleotides (nt) in sizes and are encoded by eukaryotic nuclear DNA in plants and animals. It is known that miRNAs play roles for gene expression regulation [12]. The miRNAs silence the target gene by binding to the complementary sequences of the target gene [13, 14]. In plants, the miRNAs act as important regulators of plant development and other important cellular processes [15–19]. Furthermore, many previous studies have reported that involvement of miRNAs for plant defense mechanisms against diverse pathogens [20–24].

So far, a large number of miRNAs have been identified by means of high-throughput sequencing, which has become commonly available and affordable for small RNA identification. In addition, there are databases for miRNAs such as miRBase (http://www.mirbase.org) containing at least 30,424 mature miRNAs from 206 species [25] and a plant specific miRNA database known as PMRD (http://bioinformatics.cau.edu.cn/PMRD) including 8,433 miRNAs derived from 121 plant species [26]. Currently, a large number of miRNAs have been identified in several model plants such as *Arabidopsis thaliana*, *Oryza sativa*, *Glycine max*, and *Medicago truncatula*.

Due to high conservancy of miRNAs in higher plants, the rice miRNAs have been firstly identified based on known *Arabidopsis* miRNAs resulting in 138 miRNAs belonging to 20 families [27]. In addition, many novel rice miRNAs have been revealed by small RNA sequencing. For instance, 23 new miRNAs associated with drought or salt stress [28] and 32 new miRNAs highly regulated by H_2_O_2_ [29] have been identified. Moreover, many novel microRNAs were also identified from various tissues such as embryogenic callus, spikelet, and pollen [28–30].

Several studies have reported rice miRNAs associated with RSV infection [30–33]. According to the previous study, RSV infection induced expression of some rice miRNA* and novel miRNAs [30]. Seven novel miRNAs related to RSV infection have been identified in rice [31]. Not only miRNAs but also target gene expression profiles upon RSV infection was investigated in rice cultivar susceptible to RSV using small RNA sequencing [32]. Moreover, a recent study conducted expression profiles for siRNAs and miRNAs in wild and transgenic anti-RSV rice plants suggesting the role of miRNAs in plant-virus interaction [33]. Therefore, it is clear that RSV stimulates expression of various miRNAs as well as target mRNAs in rice; however, nothing is known about time-course changes of miRNAs during RSV infection. Time-course data is an important component of miRNA studies since the regulation of gene expression by miRNA should be a dynamic process. Thus, it might be of interest to examine miRNAs which were regulated at different time points upon RSV infection.

In present study, we employed high-throughput sequencing technology to sequence and identify rice miRNAs at three different time points to monitor expression of miRNAs associated with RSV infection. We identified a total of 374 conserved miRNAs as well as 19 novel miRNAs and analyzed the expression of miRNAs at different time points. In addition, we predicted target genes of identified miRNAs and compared expression between miRNAs and their target genes.

## Materials and Methods

### Plant materials and RSV infection

*Oryza sativa* japonica cultivar Nipponbare, which is susceptible to RSV, was used in this study. Seeds of rice were surface sterilized and germinated. Rice seedlings were grown in a growth chamber at 28°C under a 16/8 light/dark photoperiod. Ten-day old rice seedlings were used for RSV inoculation. Viruliferous SBPHs (*Laodelphax striatellus* Fallen) carrying RSV were used to infect RSV in rice. Non-viruliferous SBPHs were used as mock-treated controls. SBPHs were kept transmitting virus for 48 h on rice seedlings and then were removed from all rice seedlings. The rice samples were harvested at 3 days post infection (dpi), 7 dpi, and 15 dpi. RSV infection was determined by RT-PCR using RSV nucleocapsid protein (NP) specific primers (forward 5′-CTAGTCATCTGCACCTTCTG-3′ and reverse 5′-ACTTACTGTGGGACTATGTT-3′).

### Small RNA library preparation and deep sequencing

Harvested leaves were used for total RNA extraction immediately using Oligotex mRNA minikit (Qiagen, Chatsworth, U.S.A.) according to manufacturer’s instruction. Same amount of RNAs from three individual samples were mixed for each condition to minimize bias between samples. A total of six small RNA libraries were constructed using TruSeq Small RNA Sample Prep Kit v2 (Illumina, San Diego, U.S.A.) according to manufacturer’s instruction. Polyacrylamide gel electrophoresis (PAGE) gel was used to purify small RNAs (less than 30 nt) and adapters were ligated to the 5′ and 3′ ends and then converted to cDNA by RT-PCR using Superscript II Reverse Transcriptase (Invitrogen, Carlsbad, U.S.A.). The resulting cDNAs were then amplified by PCR, gel-purified and submitted to National Instrumentation Center for Environmental Management (NICEM, Seoul, South Korea) for Illumina sequencing.

### Bioinformatic analysis to identify miRNAs

We first trimmed adapter sequences and removed poor quality sequences from the raw sequencing data using FAXTX toolkit [34]. After that, non-coding RNAs such as tRNA, rRNA, snRNA, and snoRNA were excluded using RFAM database (http://www.sanger.ac.uk/software/Rfam) by BWA program with following parameters (mismatch < = 2, gap open = 0, evalue < = 0.05) [35]. The clean read small RNA sequences were collected as fasta format files. All rice cDNA sequences were downloaded from rice genome database (http://rice.plantbiology.msu.edu/) and conserved rice mature miRNA sequences, known miRNA precursor sequences as well as known miRNA sequences from other species were downloaded from miRBase (http://www.mirbase.org/). To identify conserved and novel miRNAs in small RNAs, we used mirDeep2 program according to the program’s manual [36]. Briefly, in the first step, potential miRNA precursors were excised from the all rice cDNA using the clean read small RNA sequences as guidelines. The second step is to prepare the signature file. The bowtie-build tool is used with default options to build a Burrows-Wheeler transform index of the excised potential precursors [37]. The third step is to predict RNA secondary structures of the potential precursors. This is done with RNAfold with default parameters [38]. In the fourth step, the potential precursors were individually scored or discarded by the miRDeep2 core algorithm. Conserved mature and precursor miRNAs could be identified with estimated probability that the miRNA candidate is a true positive. Candidates with estimated probability are higher than 0.5 are regarded as novel miRNAs.

### Prediction of target mRNA for identified miRNAs

The putative target genes of miRNAs were identified by aligning mature miRNA sequences with MSU Rice Genome Annotation (version 7) using psRNA Target Server (http://plantgrn.noble.org/psRNATarget/), which is a plant small RNA target analysis server. The miRNA targets were computationally predicted as previously reported with following settings: maximum expectation: 3.0; length for complementarity scoring (hspsize): 20 bp; target accessibility: 25; flanking length around target site for target accessibility: 17 bp in upstream/13 bp in downstream and range of central mismatch leading to translational inhibition: 9–11 nt [39].

### Validation of small RNA sequencing results using quantitative real-time RT-PCR

To confirm results of miRNA expression in response to RSV infection, we selected three miRNAs including osa-MIR395y, osa-MIR167h, and osa-MIR7695. Primers for real-time RT-PCR were designed by using the miRprimer program [40]. Same total RNAs for small RNA sequencing were used for quantitative real-time RT-PCR. Gene encoding ubiquitin-5 (UBQ5) was used as a reference gene. Quantitative real-time PCR was performed with a Bio-Rad CFX384 Real-time PCR system (Bio-Rad, Hercules, U.S.A.) in Bio-Rad iQ SYBR Green Supermix (Bio-Rad) reagents according to manufacturer protocols. Detailed experimental methods were described in the previous study [41]. Data were analyzed with Bio-Rad CFX Manager software (Version 3.1).

## Results

### Identification of known small RNAs

**Fig 1:**
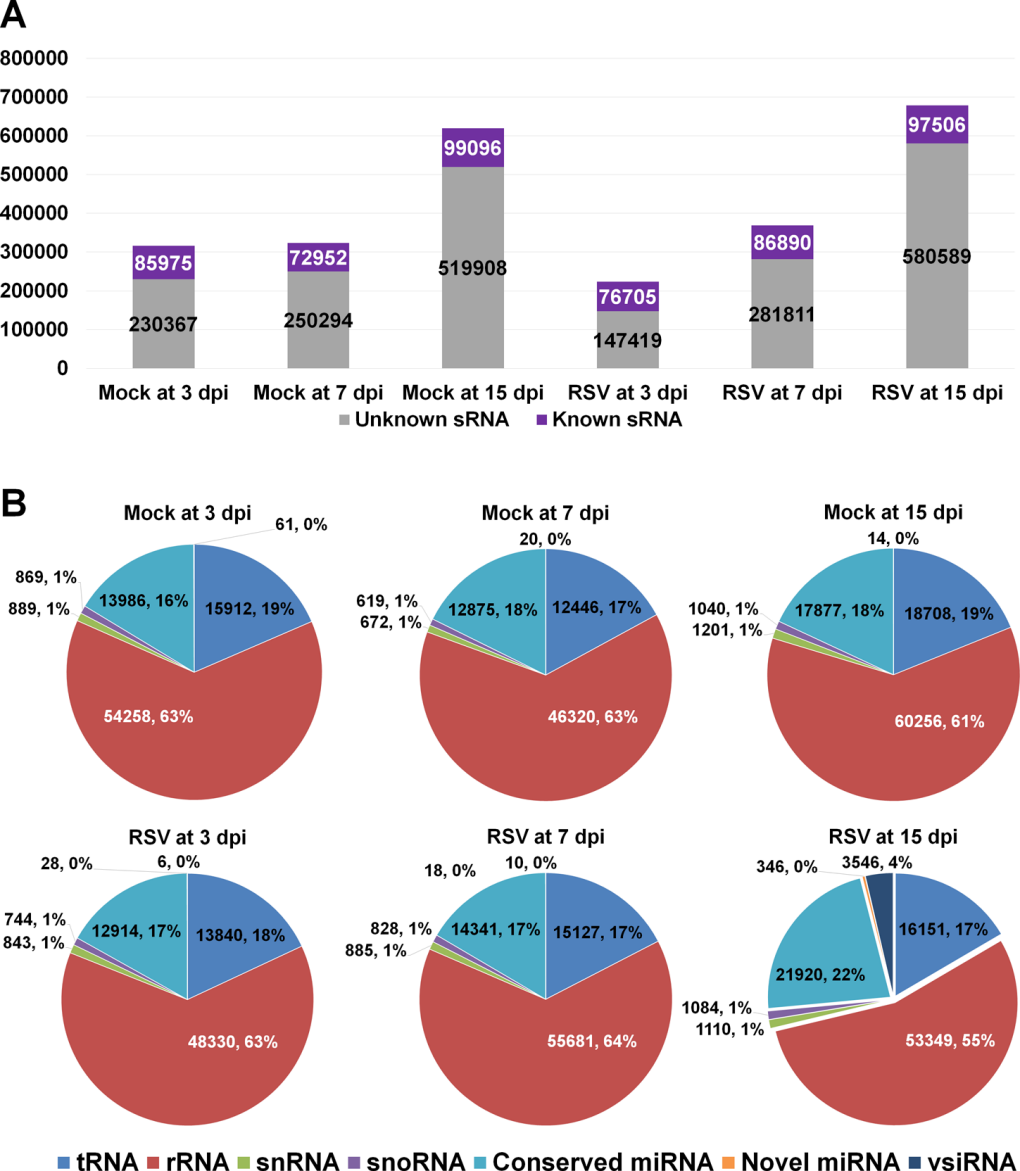
Data analysis to identify known small RNAs. (A) The numbers of sequenced small RNAs, which were further classified into known and unknown small RNAs in each sample. (B) The composition of identified known small RNAs such as tRNA, rRNA, snRNA, snoRNA, conserved miRNA, novel miRNA, and vsiRNA in each sample.

RSV-infected (RSV) and mock-treated (mock) samples at three different time points, such as 3 dpi, 7 dpi, and 15 dpi, were subjected for the construction of small RNA libraries. A total of six libraries were single-end sequenced by HiSeq 2000 system. As shown in Fig 1A, the number of sequenced reads were the highest in mock and RSV samples at 15 dpi. After processing raw data as described in materials and methods, clean reads were subjected for miRNA identification using mirDeep2 program [36]. From 5,252 to 9,588 unique reads from RSV and mock samples were mapped on the reference rice genome sequences (http://rice.plantbiology.msu.edu/). To identify known rice miRNAs, the mapped reads were used to align on known rice miRNA sequences by mirDeep2.

We identified diverse known small RNA (sRNA) such as transfer RNA (tRNA), ribosomal RNA (rRNA), small nuclear RNA (snRNA), small nucleolar RNAs (snoRNA), conserved miRNA, novel miRNA, and virus-derived small interfering RNAs (vsiRNAs). The number of identified known sRNA was the highest in mock and RSV samples at 15 dpi; however, the proportion of known sRNA was the smallest in mock (16%) and RSV (14.3%) samples at 15 dpi (Fig 1A). Of known sRNAs, rRNA was dominant followed by tRNA and conserved miRNA (Fig 1B). In addition, we identified vsiRNA (3546 reads) from the RSV sample at 15 dpi while none of vsiRNA was identified from the RSV samples at 3 and 7 dpi. The proportion of conserved miRNA at RSV sample at 15 dpi (18%) was higher than that at mock sample at 15 dpi.

### Identification of known and novel miRNAs from mock and RSV infected samples

**Fig 2:**
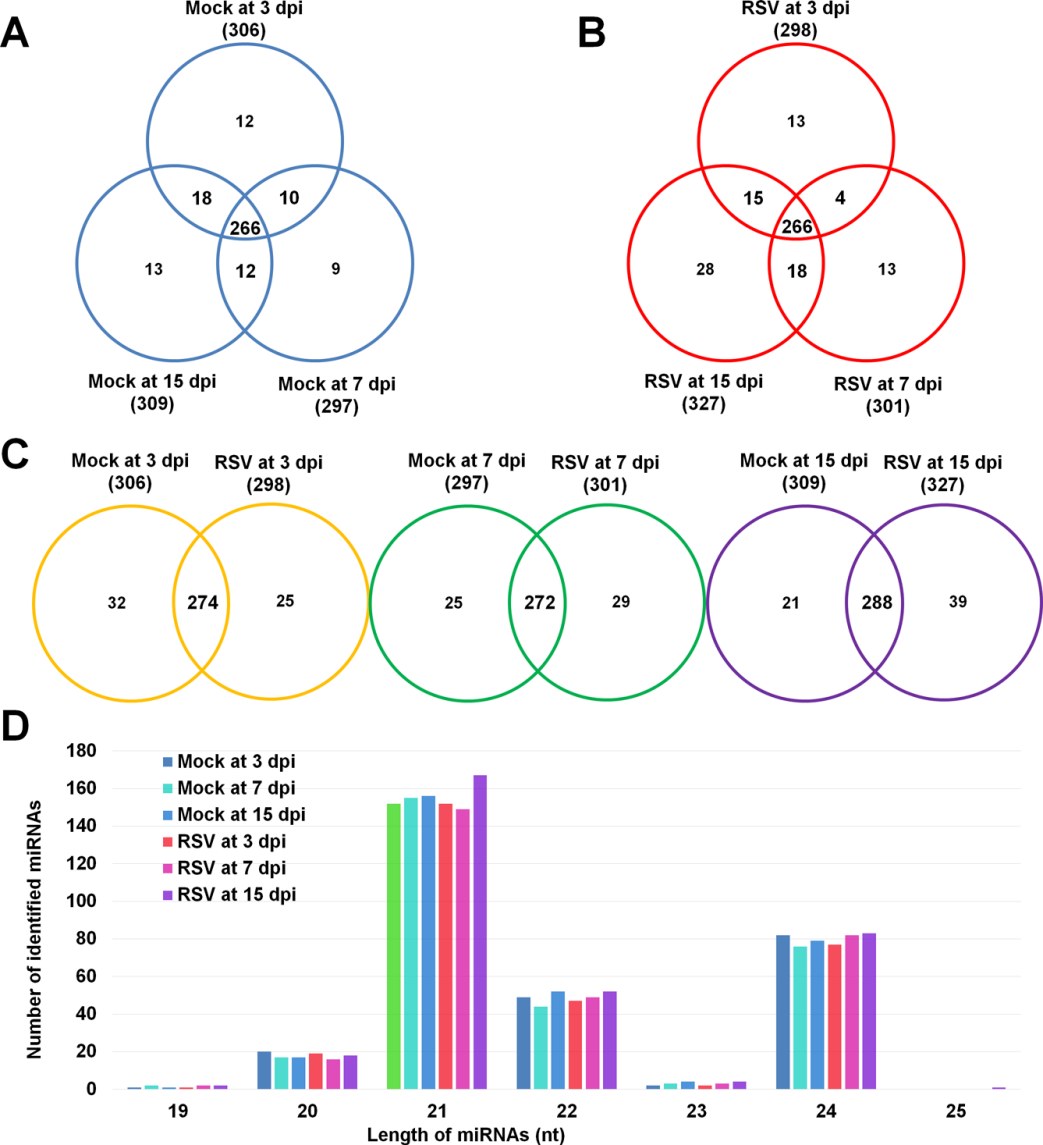
Identification of conserved miRNAs from mock and infected rice samples at different time points. (A) Venn diagram displaying the total number of identified known miRNAs in mock samples at each time point. (B) Venn diagram displaying the total number of identified known miRNAs in RSV infected samples at each time point. (C)Venn diagram displaying the total number of miRNAs identified from mock and RSV infected samples at 3 dpi, 7 dpi and 15 dpi, respectively. (D) Length distribution of identified known miRNAs identified from mock and RSV infected samples.

As a result, we identified a total of 374 known rice miRNAs from the six small RNA libraries. In detail, 306, 297, and 309 known miRNA were identified from mock samples at 3 dpi, 7 dpi and 15 dpi, respectively (Fig 2A). Furthermore, 298, 301, and 327 known miRNA were identified from RSV samples at 3 dpi, 7 dpi and 15 dpi, respectively (Fig 2B). The numbers of identified conserved miRNAs are similar among RSV and mock samples except RSV sample at 15 dpi, in which miRNA were significantly induced upon RSV infection. At least 78% (266 miRNAs) and 74% (266 miRNAs) of identified miRNAs were commonly identified in mock and RSV samples, respectively (Fig 2A and Fig 2B). In addition, 19 miRNAs were identified from only RSV sample but not mock sample. Furthermore, between mock and RSV samples (272 to 288 miRNAs), many miRNAs were commonly identified from both conditions (Fig 2C).

The size of most miRNAs ranged from 19 nt to 25 nt. Of them, 21-nt miRNAs were most abundant followed by 24-nt and 22-nt miRNAs (Fig 2D, S1 Table). In contrast, only few miRNAs with 19-nt, 23-nt and 25-nt size were identified (Fig 2D, S1 Table). Among identified known miRNAs, the most highly expressed miRNAs were members of osa-MIR 166 family, such as osa-MIR166d, osa-MIR166b, osa-MIR166c, and osa-MIR166j. They are one of well known miRNA families, which are highly conserved in plant genomes [18]. The miRNA with the highest read number was osa-MIR166d in RSV sample at 15 dpi (1,644 reads) (S1 Table).

**Fig 3:**
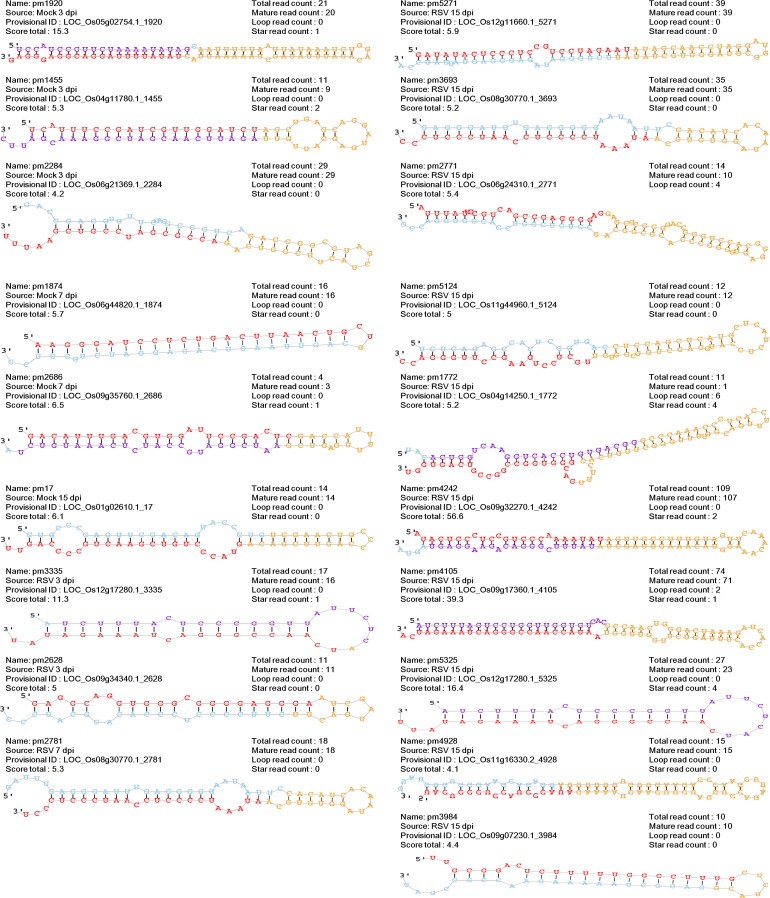
Novel 19 miRNA identified in mock and RSV infected samples at three different time points. Score total means miRDeep2 score break-down for the reported miRNA, 0.5 were used as cut-off in this study. Read counts for the mature, loop and star sequence were indicated. The predicted RNA secondary structure of the hairpin, partitioned according to miRNA biogenesis were displayed. Red, yellow, and purple colored characters indicate mature miRNAs, loop, and star miRNAs.

Furthermore, we identified a total of 19 novel miRNAs from the six samples by mirDeep2 program with the estimated probability cut-off 0.5 (Fig 3, S2 Table). The ten novel miRNAs out of 19 novel miRNAs were identified from RSV sample at 15 dpi; however, only three, two, and one miRNAs were identified from mock samples at 3 dpi, 7 dpi and 15 dpi, respectively. In addition, only two and one miRNA were identified from RSV samples at 3 dpi and 7 dpi, respectively (Fig 3, S2 Table). Unexpectedly, none of novel miRNAs was overlapped between samples.

### Identification of time point specific miRNAs

We screened miRNAs which were preferentially identified in specific time point. For example, a miRNA was identified in the mock sample at 3 dpi but should not be identified in 7 dpi in mock sample. Based on this method, we found that 30 miRNAs were preferentially expressed at 3 dpi while 21 miRNAs were preferentially expressed at 7 dpi in mock samples (Fig 2A, S3 Table). For example, the read counts for osa-miR168a-5p and osa-miR169j were 219 counts and 17 counts, respectively, at 3 dpi but they were not detectible at 7 dpi (S3 Table). On the contrary, there is no read for osa-MIR168a and osa-MIR169m at 3 dpi but the read counts for two miRNAs were 186 and 52, respectively, at 7 dpi (S3 Table). Similarly, we identified 18 miRNAs and 30 miRNAs, which were preferentially expressed at 7 dpi and 15 dpi in mock rice samples. We also identified miRNAs, which were preferentially expressed at specific time point in RSV samples. For instance, 31 miRNAs were identified but 28 miRNA were not detected between 3 dpi and 7 dpi while 43 miRNA were expressed but 17 miRNA were not detected between 7 dpi and 15 dpi in RSV samples (S3 Table). Interestingly, osa-MIR169m, osa-miR169j, osa-MIR5143b, osa-MIR5143b, osa-MIR2879, and osa-MIR2118q displays similar expression profiles in both RSV and mock samples (S3 Table).

**Fig 4:**
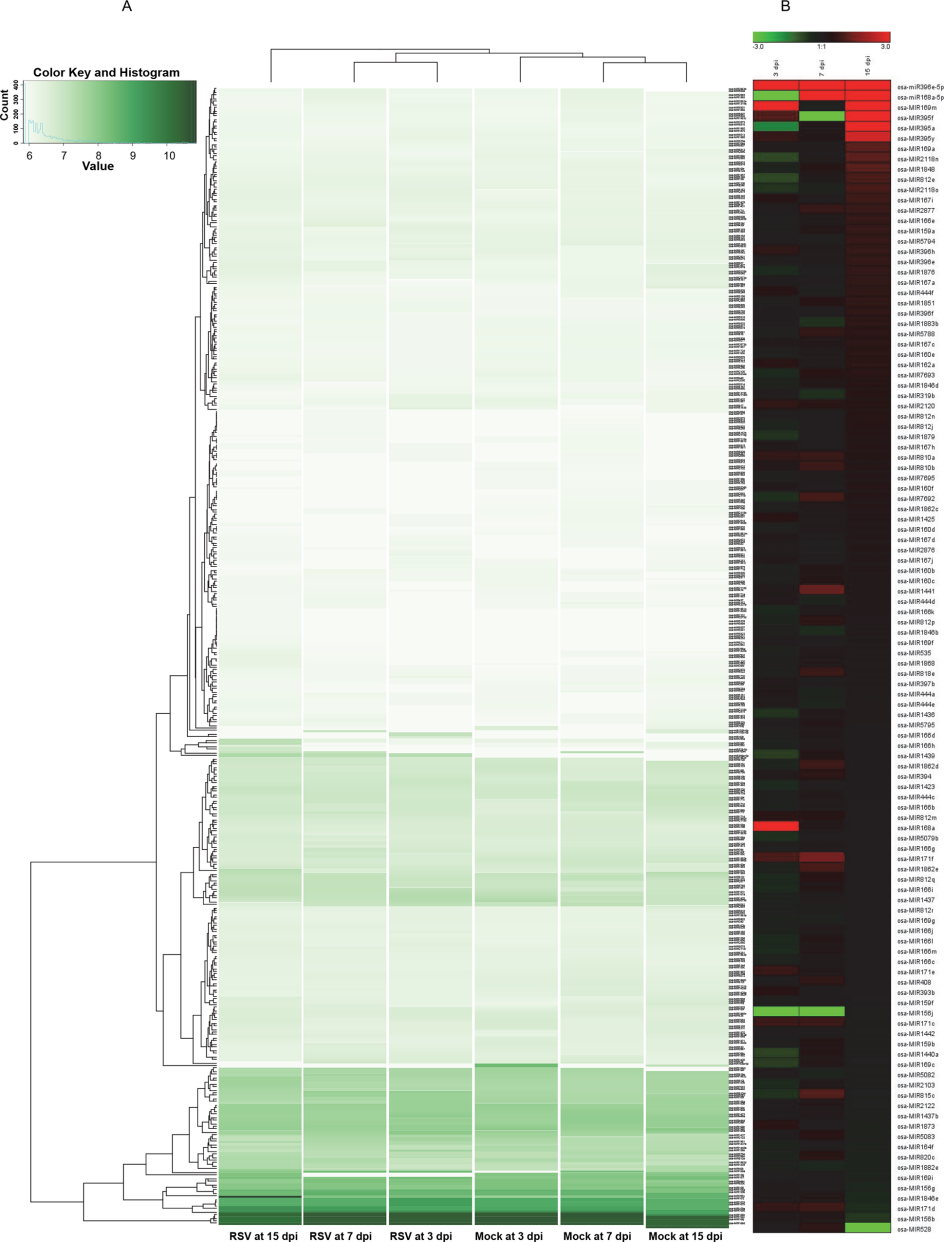
Expression profiles of 112 selected miRNAs in this study. (A) Heatmap displays relative expression of 112 miRNAs at six different samples. The miRNA read count at each sample was normalized and visualized by the DESeq package implemented in R program. (B) Heatmap displays expression of 112 miRNAs expression at the three time points upon RSV infection. The expression fold changes were calculated by comparing the mock sample to RSV infected sample at each time point. Log_2_ transformed fold changes were visualized by heatmap using genesis program. Red and green colors indicate up- and down-regulated miRNAs.

Next, we selected 112 miRNAs, in which total read counts were more than 100. The expression profiles of 112 miRNAs were analyzed at the three time points. To examine expression profiles of identified miRNAs during RSV infection, we calculated fold changes and p-values by comparing RSV sample to mock sample at each time point after normalization using DESeq package implemented in R [42]. The expression of most miRNAs were relatively stable regardless of time points. For example, the read count for osa-MIR166d in mock sample was 1,315 at 3 dpi. However, its read count was decreased to 1,177 at 7 dpi, and then again slightly increased to 1,564 at 15 dpi (Fig 4A, S1 Table).

There were only limited numbers of miRNAs, in which expression were significantly changed via time-course. For instance, the read count for osa-MIR2118n was decreased from 31 to 15 between 3 dpi and 7 dpi, respectively, while the read count for osa-MIR397b was dramatically increased from 18 reads at 7 dpi to 82 reads from 15 dpi in mock sample. The read count for osa-MIR171f was increased from 21 to 42 reads between 3 dpi and 7 dpi whereas the read count for osa-MIR395a was increased from 168 to 2,367 between 7 dpi and 15 dpi (Fig 4A, S1 Table). The read counts for many miRNAs were increased via time-course such as osa-MIR395y and osa-MIR171f in mock rice samples and osa-MIR7693 and osa-MIR159a in RSV infected samples (Fig 4A, S1 Table). Moreover, expression of most miRNAs was induced via time-course in both mock and RSV infected samples. However, we did not find any miRNA, in which expression was decreased via time-course in all samples (Fig 4A, S1 Table).

### Expression analysis of miRNA upon RSV infection at three time points

To reveal the correlation between RSV infection and miRNA expression in rice via time-course, we compared expression of miRNAs in mock to that of RSV infected sample at three time points. We found that expression of some miRNAs were specifically induced or repressed by RSV infection. A total of 32 miRNAs were down-regulated while 24 miRNAs were up-regulated upon RSV infection at 3 dpi (Fig 4B). The osa-MIR5158 (203 reads) and osa-MIR5835 (55 reads) were strongly down-regulated by RSV infection; however, osa-MIR168a (219 reads) and osa-MIR820c (163 reads) were up-regulated upon RSV infection between 3 dpi and 7 dpi (S4 Table). Twenty-five and 20 miRNAs were down-regulated but 29 and 38 miRNAs were up-regulated by RSV infection between 7 dpi and 15 dpi (Fig 4B). Interestingly, osa-MIR5835, osa-MIR399c, osa-MIR399w, and osa-MIR399g were consistently up-regulated by RSV at three time points suggesting their possible functional role in RSV infection (S4 Table).

Next, we compared the expression of 112 miRNAs, in which total reads were more than 100, upon RSV infection via time-course. Among 112 miRNAs, 21 miRNAs were significantly down-regulated while 14 miRNAs were significantly up-regulated upon RSV infection at 3 dpi based on fold changes 0.4 (Fig 4B). The osa-MIR171f and osa-MIR395f were the most up-regulated miRNAs and their expression was increased up to almost two times. On the contrary, the osa-MIR395a and osa-MIR2118n were the most down-regulated miRNAs, in which reads were decreased from 6 to 2 and 31 to 17, respectively, at 3 dpi (Fig 4B). Many numbers of miRNAs were up-regulated and relatively few miRNA were down-regulated upon RSV infection via time-course. For instance, 24 and 30 miRNAs were up-regulated while four and three miRNAs were significantly decreased at 7 dpi and 15 dpi, respectively (Fig 4B). In detail, osa-MIR1883b, osa-MIR319b, osa-MIR1846b, and osa-MIR1882e were down-regulated at 7 dpi. However, many miRNAs were up-regulated, for example, osa-MIR171f and osa-MIR1441, were the most strongly up-regulated miRNAs, in which the read count were 16 to 42 and 21 to 48, respectively, at 7 dpi (Fig 4B). Similarly, only osa-MIR156b, osa-MIR171d, and osa-MIR1846e were down-regulated by RSV infection at 15 dpi. The osa-MIR156b was strongly down-regulated (Fig 4B). On contrary, many miRNAs were highly up-regulated at 15 dpi. The osa-MIR395f and osa-MIR395a were the miRNAs in which expression was strongly up-regulated at 15 dpi (S5 Table).

The osa-MIR2120 was the only miRNA in which expression was constantly increased at 3 dpi, 7 dpi and 15 dpi; however, among 112 miRNAs, there is no miRNA in which expression was constantly suppressed at three time points (Fig 4B). Interestingly, osa-miR396e-5p was a miRNA was up-regulated by RSV infection but was not expressed in three mock samples. In addition, expression of many miRNAs was constantly changed at three time points. For example, osa-MIR171d was up-regulated at 3 dpi and 7 dpi while its expression was suppressed at 15 dpi. The osa-MIR7693c was down-regulated at 3 dpi but was up-regulated at both 7 dpi and 15 dpi (Fig 4B).

### Identification of target genes for identified miRNAs

It is of interest to find the target genes for each mRNA to elucidate functions of individual miRNAs. For that, we used the web based psRNA target server. We selected 49 miRNAs which were highly up-regulated or down-regulated by RSV infection. They include 20 miRNAs with log_2_ fold change more than 0.4, ten miRNAs which were specifically up-regulated or down-regulated by RSV infection, and 19 novel miRNAs (S6 Table). *Oryza sativa* japonica transcript library of MSU Rice Genome Annotation (version 7) was used as the database for miRNA target prediction. In total, we identified 399 predicted target rice genes including 329 unique genes after removing redundant genes. They mainly function in signal transduction, biology process, plant growth and development and especially response to endogenous stimulus (Table 1 and S7 Table). A previous study demonstrated that the miRNA156 and miRNA 159 families target transcription factors such as OsSPL2—SBP-box gene family and MYB family [18]. Again, we confirmed that those genes were targets of miRNA156 and miRNA 159 families in this study. Based on known functions for target genes, we supposed that the identified miRNAs play important roles during rice development and responses to stress. Most miRNAs had more than one target genes. For example, osa-miR396e-5p had 21 target genes including SAC9 protein and growth-regulating factor (S7 Table), which suggests that one miRNA might have several functions in rice.

**Table 1:** Rice resistance genes, which might be targets of identified miRNAs in our study.

miRNA Accession	Target Accession	Expectation	Target start	Target end	miRNA aligned fragment	Target aligned fragment	Inhibition	Target Description
osa-MIR2118n	Os08g42700.1	1.5	1457	1477	UUCCCGAUGCCUCCCAUUCCU	GGGAAUGGGUGGCAUUGGGAA	Cleavage	cDNA|resistance protein
osa-MIR2118n	Os01g05600.1	2	585	605	UUCCCGAUGCCUCCCAUUCCU	GGGGAUGGGAGGCAUCGGUAA	Cleavage	cDNA|NBS-LRR disease resistance protein
osa-MIR2118n	Os01g05620.1	2	588	608	UUCCCGAUGCCUCCCAUUCCU	GGGGAUGGGAGGCAUCGGUAA	Cleavage	cDNA|NBS-LRR disease resistance protein
osa-MIR2118n	Os01g20720.1	2.5	638	659	UUCCCGAUGCCUCCCAUUCCUA	UGGCAAUGGGAGGCAUGGGGAA	Cleavage	cDNA|CC-NBS-LRR
osa-MIR2118n	Os12g37290.1	2	1257	1277	UUCCCGAUGCCUCCCAUUCCU	GGGAAUGGGUGGUAUUGGGAA	Cleavage	cDNA|resistance protein T10rga2-1A
osa-MIR2118n	Os04g30690.1	2	749	770	UUCCCGAUGCCUCCCAUUCCUA	UUGGCAUGGGGGGCGUCGGGAA	Cleavage	cDNA|NBS type disease resistance protein
osa-MIR2118n	Os04g30610.1	2	497	518	UUCCCGAUGCCUCCCAUUCCUA	UUGGCAUGGGAGGUGUCGGGAA	Cleavage	cDNA|disease resistance protein RGA2
osa-MIR2118n	Os04g30660.1	2	749	770	UUCCCGAUGCCUCCCAUUCCUA	UUGGUAUGGGCGGCAUCGGGAA	Cleavage	cDNA|NBS type disease resistance protein
osa-MIR2118n	Os01g72390.1	3	425	446	UUCCCGAUGCCUCCCAUUCCUA	UGGGGAUGGGUGGGAUUGGGAA	Translation	cDNA|NBS type disease resistance protein
osa-MIR2118q	Os01g05600.1	2.5	585	605	UUCCCGAUGCCUCCUAUUCCU	GGGGAUGGGAGGCAUCGGUAA	Cleavage	cDNA|NBS-LRR disease resistance protein
osa-MIR2118q	Os01g05620.1	2.5	588	608	UUCCCGAUGCCUCCUAUUCCU	GGGGAUGGGAGGCAUCGGUAA	Cleavage	cDNA|NBS-LRR disease resistance protein
osa-MIR2118q	Os01g20720.1	3	638	659	UUCCCGAUGCCUCCUAUUCCUA	UGGCAAUGGGAGGCAUGGGGAA	Cleavage	cDNA|CC-NBS-LRR
osa-MIR2118q	Os12g37290.1	2.5	1257	1277	UUCCCGAUGCCUCCUAUUCCU	GGGAAUGGGUGGUAUUGGGAA	Cleavage	cDNA|resistance protein T10rga2-1A
osa-MIR2118q	Os04g30690.1	2.5	749	770	UUCCCGAUGCCUCCUAUUCCUA	UUGGCAUGGGGGGCGUCGGGAA	Cleavage	cDNA|NBS type disease resistance protein
osa-MIR2118q	Os04g30610.1	2.5	497	518	UUCCCGAUGCCUCCUAUUCCUA	UUGGCAUGGGAGGUGUCGGGAA	Cleavage	cDNA|disease resistance protein RGA2
osa-MIR2118q	Os04g30660.1	2.5	749	770	UUCCCGAUGCCUCCUAUUCCUA	UUGGUAUGGGCGGCAUCGGGAA	Cleavage	cDNA|NBS type disease resistance protein
osa-MIR2118q	Os08g42700.1	2	1457	1477	UUCCCGAUGCCUCCUAUUCCU	GGGAAUGGGUGGCAUUGGGAA	Cleavage	cDNA|resistance protein
osa-MIR395y	Os11g44580.1	3	1171	1190	GUGAAGUGUUUGGGGGAACU	GGUGCCUCCGAAUACUUCAC	Cleavage	cDNA|go35 NBS-LRR
osa-MIR399g	Os05g30220.1	2.5	1680	1700	UGCCAAAGGAGAUUUGCCCAG	CUUGGAAGAUCUCCUUUGGCA	Cleavage	cDNA|disease resistance RPP13-like protein 1
osa-MIR399g	Os11g45050.1	3	1879	1899	UGCCAAAGGAGAUUUGCCCAG	UUGCGCAGACUUCCUUUGGCA	Cleavage	cDNA|NBS-LRR disease resistance protein
osa-MIR399g	Os11g44960.1	3	1882	1902	UGCCAAAGGAGAUUUGCCCAG	UUGCGCAGACUUCCUUUGGCA	Cleavage	cDNA|NBS-LRR disease resistance protein
pm4242	Os12g18374.2	1	3596	3618	AUACUCCCUCCGUCCCAAAAUAU	AUAUUUUGGGACGGAAGGAGUAU	Cleavage	cDNA|NB-ARC domain containing protein
pm5124	Os11g45190.1	1.5	3533	3553	UGCUCCUGAAGCCUUGGGACC	GGUCCCAGGGCUUGAGGAGCA	Cleavage	cDNA|NB-ARC domain containing protein

There were 15 disease resistance genes, which were predicted as targets of miRNAs including osa-MIR2118n, osa-MIR2118q, osa-MIR395y, osa-MIR399g and two newly identified miRNAs, pm4242 and pm5124 (Table 1). They were all up-regulated at 15 dpi. There were nine target genes for osa-MIR2118n and osa-MIR2118q and eight genes of them were targeted by the two miRNAs. Interestingly, both miRNAs were down-regulated at 3 dpi but were highly down-regulated at 15 dpi by RSV infection. Furthermore, osa-MIR395y targeting a NBS-LRR gene was up-regulated at 3 dpi and 15 dpi by RSV infection. The osa-MIR399g targeting three NBS-LRR genes was specifically up-regulated by RSV at 15 dpi. In addition, novel two miRNAs such as pm4242 and pm5124 identified from RSV infected sample at 15 dpi, target a NB-ARC domain gene (Table 1). However, the results from the prediction program should be validated by other experimental approaches in near future.

### Comparative analysis of expression of miRNA and their target genes upon RSV infection

The target RNA expression is suppressed by cleaving of miRNA or suppressing translation [43]. Previously, we conducted rice transcriptome analysis upon RSV infection at 3 dpi, 7 dpi and 15 dpi by RNA-Seq [41]. For small RNA sequencing, we used same plant materials which were used for RNA-Seq. To find the correlation between miRNA expression and their target gene expression, we derived the 399 rice genes expression data, which were predicted as targets of the 30 known miRNAs and 19 newly identified miRNAs. Subsequently, expression of miRNA and their predicted targets were compared (S7 Table). Unfortunately, we did find any negative correlation for expression between miRNAs and their target genes. For example, osa-MIR820c targeting five genes, was significantly up-regulated by RSV infection at 7 dpi. Of them, four genes including Os05g00996.1, Os10g42196.1, Os11g03310.1, and Os11g13650.1 were all down-regulated except Os03g02010.4 displaying no change in gene expression at 7 dpi. The osa-MIR166k was down-regulated at 3 dpi and nine of 13 target genes for osa-MIR166k were up-regulated. The osa-MIR5158 was down-regulated by RSV infection at 3 dpi, and its three target genes such as Os03g17980.1, Os03g17980.2 and Os08g37800.1, were up-regulated (S7 Table). According to our results, expression of many miRNAs were positively correlated with expression of their target genes. For example, osa-MIR167i and osa-MIR169a were all up-regulated upon RSV infection, but most predicted targets were also up-regulated at 15 dpi (S7 Table). Moreover, expression of many miRNAs affects negatively or positively expression of their target genes (S7 Table).

**Fig 5:**
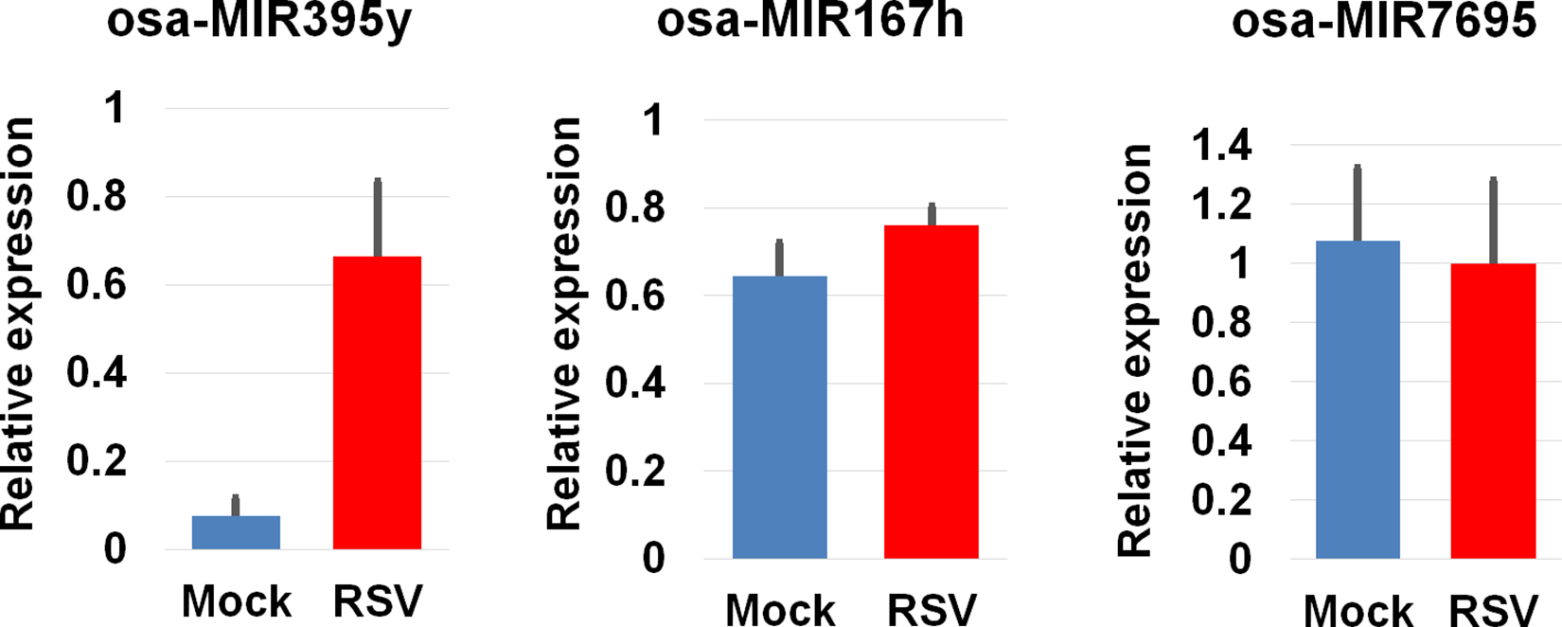
Expression of three selected miRNAs by quantitative real-time RT-PCR. Relative expression of three selected miRNAs including osa-MIR395y, osa-MIR167h, and osa-MIR7695 was determined by quantitative real-time RT-PCR. Total RNAs from mock and RSV samples at 15 dpi were used. Black colored bar indicates the standard deviation.

We validated expression of the three selected miRNAs between mock and RSV samples at 15 dpi. by quantitative real-time RT-PCR (Fig 5). Expression of two miRNAs (osa-MIR395y and osa-MIR167h) was up-regulated by quantitative real-time RT-PCR, in which results were consistent with those by small RNA sequencing. In case of osa-MIR7695, the expression of this miRNA between mock and RSV samples was very similar although the result of small RNA sequencing showed slight up-regulation of osa-MIR7695 by RSV infection.

## Discussion

Over the past decade, a large number of miRNAs have been identified and it is clear that miRNAs play critical roles on many biological processes or defense mechanisms at the post-transcription stage in plants [18, 23]. High-throughput sequencing becomes commonly available and affordable for small RNA identification.

Although there were several studies performing rice miRNA expression upon RSV infection [30–33], our study shows more dynamic information of miRNA expression upon RSV infection via time-course. We identified 374 known rice miRNAs, which cover 52.5% (374/713) of known rice miRNA in miRBase and the number of identified miRNAs were similar as compared to previous reports [30, 44]. Moreover, 19 novel miRNAs were also identified from the six samples. The number of identified miRNAs might be dependent on several factors such as sample conditions, sequencing methods, and bioinformatic tools. For example, a recent study using six libraries revealed 570 miRNAs [32] while our study using six libraries identified 374 libraries. The recent study used a single time point (7 dpi) [3] while we harvested samples at three different time points. It might be of interest to compare the list of miRNAs between two studies at 7 dpi; however, the complete list of miRNAs in the previous study is not available. Based on results from this study and a previous study [30], we supposed that the number of identified miRNAs was increased when the samples were harvested at the late stages such as 15 days (this study) and 3 weeks (a previous study) after RSV infection. In other studies using samples from 7 dpi after RSV infection [31, 32], the numbers of identified miRNAs were less than that of our study. The difference of identified miRNAs might be caused by different sample conditions or the bioinformatic program to predict miRNAs [33].

The previous study using samples at 7 dpi after RSV infection successfully mapped vsiRNA on the four RSV RNA genomes [45]; however, we did not obtain vsiRNAs from the RSV infected samples at 3 and 7 dpi. We only identified vsiRNAs from RSV at 15 dpi after RSV infection. This result indicates that the virus infection time might be an important factor to produce vsiRNAs in the RSV infected plants.

Time-course analysis confirmed that the list of many expressed miRNAs at three time points were quite similar. For example, the well-known plant miRNAs such as osa-MIR166, osa-MIR167 and osa-MIR395 families are known to be involved in early rice developmental stages [18]. This result suggest that the steady-state levels of miRNAs need to be properly controlled to ensure normal rice development [44]. However, some miRNAs were strongly up-regulated in mock and RSV infected samples at 15 dpi. The up-regulation of miRNA expression might be caused by the exogenous stresses, such as RSV infection which resulted in high level of RSV replication in RSV infected sample. A previous report also demonstrated that miRNAs families 156, 164, 165, and 167, were highly accumulated in *Tobacco mosaic virus* infected *Nicotiana tabacum* as compared to non-infected tissues [46]. Similarly, osa-MIR167 family reported as a known miRNA and functions in plant development [18, 46], was highly up-regulated upon RSV infection. Some miRNAs had a short life time, such as 12 miRNAs were not expressed between 3 dpi and 7 dpi in mock rice. It seems that a miRNA degradation pathway might function in this phase. Unfortunately, there is no study reporting on miRNA degradation mechanism in rice. However, a family of exoribonucleases encoded by the small RNA degrading nuclease (SDN) genes in *Arabidopsis* degrade mature miRNAs [47]. In addition, there were some rice miRNAs were up-regulated at the late stage. It is logical that miRNAs are produced when they are induced by several stimuli and can be degraded when they are useless any more during plant development.

Many identified miRNAs in this study were also identified in previous reports such as osa-MIR166 family and osa-MIR167 family and their read counts were very high [30, 31, 44]. It is possible that the frequently identified miRNAs might be multifunctional and important for rice development. We also identified miRNAs which highly regulated by RSV infection at three time points. About 12% (14/112) miRNAs were up-regulated while about 20% (21/112) miRNAs were down-regulated. However, this expression pattern was dramatically changed at 7 dpi and 15 dpi. There were about 21% (24/112) and 30% (30/112) miRNAs, which were up-regulated while only few numbers of miRNAs were down-regulated. The number of up-regulated miRNAs was similar both at 3 dpi and 7 dpi but the number of up-regulated miRNAs was dramatically increased from 7 dpi to 15 dpi. Many miRNAs were up-regulated by RSV at the late stage when a large amount of RSV RNAs had been accumulated. However, transcripts for the down-regulated miRNAs were strongly decreased via time-course. This result demonstrates that many miRNAs become very active in rice in response to RSV infection. A previous report also demonstrated that several rice miRNAs are involved in different virus infection [30]. In our study, we revealed some miRNAs were specifically regulated by RSV. For instance, osa-MIR2118q, osa-MIR395w, osa-MIR399c, osa-MIR399g, osa-MIR5158, and osa-MIR5835 were specifically up-regulated by RSV infection at different time point, respectively. They might play important roles in RSV infection. Moreover, osa-miR396e-5p was up-regulated by RSV at all stages. This miRNA was a member of osa-miR396 family containing several members. It might be valuable to characterize the role of osa-miR396e-5p associated with RSV infection in near future. Although many numbers of miRNAs associated with RSV infections were identified, the characterized miRNA associated with RSV infection is limited. For example, a recent study showed that monocot specific miR444 is required for the antiviral signaling from virus infection to OsRDR1 expression [48].

The target RNA is cleaved by complementarity or translational suppression to their target [43, 49]. We found that 399 rice genes were targets of selected 49 miRNAs. Similar with a previous report, those target genes were mainly associated with signal transduction, retrotransposon protein, transcription factor, biological functions, differentiation, and disease resistance [44]. Based on these results, RSV infection might suppress genes associated with rice growth and development and interrupt defense mechanism against viruses resulting in viral disease symptoms [3]. Interestingly, 15 disease resistance genes were predicted as targets of miRNAs, such as osa-MIR2118n, osa-MIR2118q, osa-MIR395y, and osa-MIR399g as well as two newly identified miRNAs, pm4242 and pm5124. Those miRNAs were significantly up-regulated upon RSV infection except osa-MIR399g, in which transcript was not expressed at 15 dpi. It is well known that members in miR2118 family target NBS-LRR containing gene families in many plant species. In rice, there are 18 pre-miRNA variants correspond to 16 different mature miRNAs [50, 51]. The miR482 and miR2118 were assigned into miR482/2118 superfamily and target the coding sequence for the P-loop motif in disease resistance genes possessing nucleotide binding site (NBS) and leucine-rich repeat (LRR) motifs in various species [52, 53]. A previous report demonstrated that miR482 targets mRNAs with coiled-coil domains causing mRNA decay and production of secondary siRNAs in a manner that depends on RNA-dependent RNA polymerase 6 in *Nicotiana benthamiana* [53]. Although there is no report on function pathway of osa-MIR2118 in rice, some miRNAs might play a conserved role in NB-LRR/LRR mediated gene regulation and pathogen resistance [21]. In addition, osa-MIR395 was frequently up-regulated upon environmental stresses [54]. MIR399 involved in the regulation of the cellular response [55] was down regulated upon RSV infection in this study. It might be worth of studying on the mechanism how those miRNAs regulate host gene expression upon RSV infection.

Suppression of the target gene by miRNAs is an essential step for gene silencing. For example, up-regulation of osa-MIR820c led to down-regulation of target genes. Nevertheless, only a small set of target genes were down-regulated by miRNAs in this study. Similar results were also reported in rice samples infected *Magnaporthe oryzae* [56]. It seems that there might be time lags of expression between miRNA and their target genes [57]. Moreover, it is also evident that host mRNA gene expression were not solely affected by miRNAs but also there are several unknown factors which regulate gene expression in plants [58]. We also carefully suppose that expression of a target gene which was suppressed by miRNA can be recovered by unknown other signal pathway.

In summary, we investigated miRNAs expression upon RSV infection via time-course. Several crucial miRNAs related to RSV infection were identified including a total of 15 disease resistance genes. Our results provide a list of known and novel miRNAs which were involved in RSV infection. The identified miRNAs are good candidates to study novel insight into the dynamic profiles of miRNAs in RSV infection and contribute to the understanding of the regulatory roles of miRNAs via time-course.

## Supporting Information

S1 Table Identified known miRNAs in mock and RSV infected rice samples at three different time points. (XLSX)

S2 Table Identified novel miRNAs in mock and RSV infected rice samples at three different time points. (XLSX)

S3 Table Time specific miRNAs in mock and RSV infected rice samples. (XLSX)

S4 Table RSV infection associated miRNAs in mock and RSV infected samples. (XLSX)

S5 Table Expression profiles of miRNAs upon RSV infection at three time points. (XLSX)

S6 Table Selected miRNAs used for expression profiles of target genes. (XLSX)

S7 Table Predicted targets of identified miRNAs. (XLSX)

## References

[1] Goff SA, Ricke D, Lan T-H, Presting G, Wang R, Dunn M, et al A draft sequence of the rice genome (*Oryza sativa* L. ssp. *japonica*). Science. 2002; 296:92–100. 11935018 10.1126/science.1068275

[2] Yu J, Hu S, Wang J, Wong GK-S, Li S, Liu B, et al A draft sequence of the rice genome (*Oryza sativa* L. ssp. *indica*). Science. 2002; 296:79–92. 11935017 10.1126/science.1068037

[3] Cho WK, Lian S, Kim S-M, Park S-H, Kim K-H. Current insights into research on rice stripe virus. Plant Pathology J. 2013; 29:30–40. 10.5423/PPJ.RW.10.2012.0158 PMC4174810 25288949

[4] Hibino H. Biology and epidemiology of rice viruses. Annu Rev Phytopathol. 1996; 34:249–274. PubMed Central PMCID: PMC15012543. 15012543 10.1146/annurev.phyto.34.1.249

[5] Wei T-Y, Yang J-G, Liao F-L, Gao F-L, Lu L-M, Zhang X-T, et al Genetic diversity and population structure of rice stripe virus in China. J Gen Virol. 2009; 90:1025–1034. 10.1099/vir.0.006858-0 19264655

[6] He D-C, Zhan J, Cheng Z-B, Xie L-H. Viruliferous rate of small brown planthopper is a good indicator of rice stripe disease epidemics. Sci Rep. 2016; 6:21376 10.1038/srep21376 26898155 PMC4761966

[7] Shiba T, Hirae M, Hayano-Saito Y, Uematsu H, Sasaya T, Higuchi H, et al Seasonal changes in the percentage of rice stripe virus viruliferous *Laodelphax striatellus* (Hemiptera: Delphacidae) in paddy fields in Japan. J Eco Entomol. 2016: tow061. 10.1093/jee/tow061 27099363

[8] Toriyama S, Takahashi M, Sano Y, Shimizu T, Ishihama A. Nucleotide sequence of RNA 1, the largest genomic segment of rice stripe virus, the prototype of the *tenuiviruses*. J Gen Virol. 1994; 75:3569–3579. PubMed Central PMCID: PMC7996149. 7996149 10.1099/0022-1317-75-12-3569

[9] Takahashi M, Toriyama S, Hamamatsu C, Ishihama A. Nucleotide sequence and possible ambisense coding strategy of rice stripe virus RNA segment 2. J Gen Virol. 1993; 74:769–773. PubMed Central PMCID: PMC8468559. 8468559 10.1099/0022-1317-74-4-769

[10] Hayano Y, Kakutani T, Hayashi T, Minobe Y. Coding strategy of rice stripe virus: major nonstructural protein is encoded in viral RNA segment 4 and coat protein in RNA complementary to segment 3. Virology. 1990; 177:372–374. PubMed Central PMCID: PMC2141205. 2141205 10.1016/0042-6822(90)90493-b

[11] Kakutani T, Hayano Y, Hayashi T, Minobe Y. Ambisense segment 3 of rice stripe virus: the first instance of a virus containing two ambisense segments. J Gen Virol. 1991; 72:465–468. PubMed Central PMCID: PMC1993885. 1993885 10.1099/0022-1317-72-2-465

[12] Chen K, Rajewsky N. The evolution of gene regulation by transcription factors and microRNAs. Nat Rev Genet. 2007; 8:93–103. 17230196 10.1038/nrg1990

[13] Bartel DP. MicroRNAs: target recognition and regulatory functions. Cell. 2009; 136:215–233. 10.1016/j.cell.2009.01.002 19167326 PMC3794896

[14] Kusenda B, Mraz M, Mayer J, Pospisilova S. MicroRNA biogenesis, functionality and cancer relevance. Biomed Papers. 2006; 150:205–215. 10.5507/bp.2006.029 17426780

[15] Wang J-W, Wang L-J, Mao Y-B, Cai W-J, Xue H-W, Chen X-Y. Control of root cap formation by microRNA-targeted auxin response factors in *Arabidopsis*. Plant Cell. 2005; 17:2204–2216. 16006581 10.1105/tpc.105.033076 PMC1182483

[16] Palatnik JF, Allen E, Wu X, Schommer C, Schwab R, Carrington JC, et al Control of leaf morphogenesis by microRNAs. Nature. 2003; 425:257–263. 12931144 10.1038/nature01958

[17] Kidner CA, Martienssen RA. The developmental role of microRNA in plants. Curr Opin Plant Biol. 2005; 8:38–44. 15653398 10.1016/j.pbi.2004.11.008

[18] Jones-Rhoades MW, Bartel DP, Bartel B. MicroRNAs and their regulatory roles in plants. Annu Rev Plant Biol. 2006; 57:19–53. 16669754 10.1146/annurev.arplant.57.032905.105218

[19] Guo H-S, Xie Q, Fei J-F, Chua N-H. MicroRNA directs mRNA cleavage of the transcription factor NAC1 to downregulate auxin signals for *Arabidopsis* lateral root development. Plant Cell. 2005; 17:1376–86. 15829603 10.1105/tpc.105.030841 PMC1091761

[20] Simón-Mateo C, García JA. MicroRNA-guided processing impairs plum pox virus replication, but the virus readily evolves to escape this silencing mechanism. J Virol. 2006; 80:2429–2436. 16474149 10.1128/JVI.80.5.2429-2436.2006 PMC1395392

[21] Li F, Pignatta D, Bendix C, Brunkard JO, Cohn MM, Tung J, et al MicroRNA regulation of plant innate immune receptors. Proc Natl Acad Sci USA. 2012; 109:1790–1795. 10.1073/pnas.1118282109 22307647 PMC3277104

[22] Padmanabhan C, Zhang X, Jin H. Host small RNAs are big contributors to plant innate immunity. Curr Opin Plant Biol. 2009; 12:465–472. 10.1016/j.pbi.2009.06.005 19608454

[23] Gan Q-h, Chi X-y, Qin S. Roles of microRNA in plant defense and virus offense interaction. Plant Cell Rep. 2008; 27:1571–1579. 10.1007/s00299-008-0584-z 18626646

[24] Navarro L, Dunoyer P, Jay F, Arnold B, Dharmasiri N, Estelle M, et al A plant miRNA contributes to antibacterial resistance by repressing auxin signaling. Science. 2006; 312:436–439. 16627744 10.1126/science.1126088

[25] Griffiths-Jones S, Grocock RJ, Van Dongen S, Bateman A, Enright AJ. miRBase: microRNA sequences, targets and gene nomenclature. Nucl Acids Res. 2006; 34:D140–D144. 16381832 10.1093/nar/gkj112 PMC1347474

[26] Zhang Z, Yu J, Li D, Zhang Z, Liu F, Zhou X, et al PMRD: plant microRNA database. Nucl Acids Res. 2010; 38:D806–D813. 10.1093/nar/gkp818 19808935 PMC2808885

[27] Jones-Rhoades MW, Bartel DP. Computational identification of plant microRNAs and their targets, including a stress-induced miRNA. Mol Cell. 2004; 14(6):787–799. 15200956 10.1016/j.molcel.2004.05.027

[28] Sunkar R, Zhou X, Zheng Y, Zhang W, Zhu J-K. Identification of novel and candidate miRNAs in rice by high throughput sequencing. BMC Plant Biol. 2008; 8:25 10.1186/1471-2229-8-25 18312648 PMC2292181

[29] Wei LQ, Yan LF, Wang T. Deep sequencing on genome-wide scale reveals the unique composition and expression patterns of microRNAs in developing pollen of *Oryza sativa*. Genome Biol. 2011; 12:R53 10.1186/gb-2011-12-6-r53 21679406 PMC3218841

[30] Du P, Wu J, Zhang J, Zhao S, Zheng H, Gao G, et al Viral infection induces expression of novel phased microRNAs from conserved cellular microRNA precursors. PLoS Pathog. 2011; 7:e1002176 10.1371/journal.ppat.1002176 21901091 PMC3161970

[31] Guo W, Wu G, Yan F, Lu Y, Zheng H, Lin L, et al Identification of novel *Oryza sativa* miRNAs in deep sequencing-based small RNA libraries of rice infected with rice stripe virus. PLoS One. 2012; 7:e46443 10.1371/journal.pone.0046443 23071571 PMC3468594

[32] Yang J, Zhang F, Li J, Chen J, Zhang H. Integrative analysis of the microRNAome and transcriptome illuminates the response of susceptible rice plants to rice stripe virus. PLoS One. 2015; 11:e0146946. 10.1371/journal.pone.0146946 PMC4723043 26799317

[33] Guo C, Li L, Wang X, Liang C. Alterations in siRNA and miRNA expression profiles detected by deep sequencing of transgenic rice with siRNA-mediated viral resistance. PLoS One. 2015; 10:e0116175 10.1371/journal.pone.0116175 25559820 PMC4283965

[34] Blankenberg D, Gordon A, Von Kuster G, Coraor N, Taylor J, Nekrutenko A. Manipulation of FASTQ data with Galaxy. Bioinformatics. 2010; 26:1783–1785. 10.1093/bioinformatics/btq281 20562416 PMC2894519

[35] Li H, Durbin R. Fast and accurate long-read alignment with Burrows–Wheeler transform. Bioinformatics. 2010; 26(5): 589–95. 10.1093/bioinformatics/btp698 20080505 PMC2828108

[36] Friedländer MR, Mackowiak SD, Li N, Chen W, Rajewsky N. miRDeep2 accurately identifies known and hundreds of novel microRNA genes in seven animal clades. Nucl Acids Res. 2012; 40:37–52. 10.1093/nar/gkr688 21911355 PMC3245920

[37] Langmead B. Aligning short sequencing reads with Bowtie. Curr Protoc Bioinformatics. 2010: 11.7.1–11.7.4. 21154709 10.1002/0471250953.bi1107s32 PMC3010897

[38] Hofacker IL. Vienna RNA secondary structure server. Nucl Acids Res. 2003; 31:3429–3431. 12824340 10.1093/nar/gkg599 PMC169005

[39] Dai X, Zhao PX. psRNATarget: a plant small RNA target analysis server. Nucl Acids Res. 2011; 39:W155–W159. 10.1093/nar/gkr319 21622958 PMC3125753

[40] Busk PK. A tool for design of primers for microRNA-specific quantitative RT-qPCR. BMC Bioinformatics. 2014; 15:1. 24472427 10.1186/1471-2105-15-29 PMC3922658

[41] Cho WK, Lian S, Kim S-M, Seo BY, Jung JK, Kim K-H. Time-course RNA-Seq analysis reveals transcriptional changes in rice plants triggered by rice stripe virus infection. PLoS One. 2015; 10:e0136736 10.1371/journal.pone.0136736 26305329 PMC4549299

[42] Anders S, Huber W. Differential expression analysis for sequence count data. Genome Biol. 2010; 11:R106 10.1186/gb-2010-11-10-r106 20979621 PMC3218662

[43] Lanet E, Delannoy E, Sormani R, Floris M, Brodersen P, Crété P, et al Biochemical evidence for translational repression by *Arabidopsis* microRNAs. Plant Cell. 2009; 21:1762–1768. 10.1105/tpc.108.063412 19531599 PMC2714937

[44] Yi R, Zhu Z, Hu J, Qian Q, Dai J, Ding Y. Identification and expression analysis of microRNAs at the grain filling stage in rice (*Oryza sativa* L.) via deep sequencing. PLoS One. 2013; 8:e57863 10.1371/journal.pone.0057863 23469249 PMC3585941

[45] Yan F, Zhang H, Adams MJ, Yang J, Peng J, Antoniw JF, et al Characterization of siRNAs derived from rice stripe virus in infected rice plants by deep sequencing. Arch Virol. 2010; 155:935–940. 10.1007/s00705-010-0670-8 20396917

[46] Bazzini A, Hopp H, Beachy R, Asurmendi S. Infection and coaccumulation of tobacco mosaic virus proteins alter microRNA levels, correlating with symptom and plant development. Proc Natl Acad Sci USA. 2007; 104:12157–12162. 17615233 10.1073/pnas.0705114104 PMC1924585

[47] Ramachandran V, Chen X. Degradation of microRNAs by a family of exoribonucleases in *Arabidopsis*. Science. 2008; 321:1490–1492. 10.1126/science.1163728 18787168 PMC2570778

[48] Wang H, Jiao X, Kong X, Humaira S, Wu Y, Chen X, et al A signaling cascade from miR444 to RDR1 in rice antiviral RNA silencing pathway. Plant Physiol. 2016; 170:2365–2377. 10.1104/pp.15.01283 26858364 PMC4825140

[49] Brodersen P, Sakvarelidze-Achard L, Bruun-Rasmussen M, Dunoyer P, Yamamoto YY, Sieburth L, et al Widespread translational inhibition by plant miRNAs and siRNAs. Science. 2008; 320:1185–1190. 10.1126/science.1159151 18483398

[50] Johnson C, Kasprzewska A, Tennessen K, Fernandes J, Nan G-L, Walbot V, et al Clusters and superclusters of phased small RNAs in the developing inflorescence of rice. Genome research. 2009; 19:1429–1440. 10.1101/gr.089854.108 19584097 PMC2720183

[51] Vogel JP, Garvin DF, Mockler TC, Schmutz J, Rokhsar D, Bevan MW, et al Genome sequencing and analysis of the model grass *Brachypodium distachyon*. Nature. 2010; 463:763–768. 10.1038/nature08747 20148030

[52] Zhai J, Jeong D-H, De Paoli E, Park S, Rosen BD, Li Y, et al MicroRNAs as master regulators of the plant NB-LRR defense gene family via the production of phased, trans-acting siRNAs. Genes Dev. 2011; 25:2540–2553. 10.1101/gad.177527.111 22156213 PMC3243063

[53] Shivaprasad PV, Chen H-M, Patel K, Bond DM, Santos BA, Baulcombe DC. A microRNA superfamily regulates nucleotide binding site–leucine-rich repeats and other mRNAs. Plant Cell. 2012; 24:859–874. 10.1105/tpc.111.095380 22408077 PMC3336131

[54] Phillips JR, Dalmay T, Bartels D. The role of small RNAs in abiotic stress. FEBS Lett. 2007; 581:3592–3597. 17451688 10.1016/j.febslet.2007.04.007

[55] Branscheid A, Sieh D, Pant BD, May P, Devers EA, Elkrog A, et al Expression pattern suggests a role of MiR399 in the regulation of the cellular response to local Pi increase during arbuscular mycorrhizal symbiosis. Mol Plant-Microbe Interact. 2010; 23:915–926. 10.1094/MPMI-23-7-0915 20521954

[56] Li Y, Lu YG, Shi Y, Wu L, Xu YJ, Huang F, et al Multiple rice miRNAs are involved in immunity against the blast fungus *Magnaporthe oryzae*. Plant Physiol. 2014; 164:1077–1092. 10.1104/pp.113.230052 24335508 PMC3912081

[57] Jayaswal V, Lutherborrow M, Ma DD, Yang YH. Identification of microRNAs with regulatory potential using a matched microRNA-mRNA time-course data. Nucl Acids Res. 2009; 37(8):e60 10.1093/nar/gkp153 19295134 PMC2677888

[58] Balmer JE, Blomhoff R. Gene expression regulation by retinoic acid. J Lipid Res. 2002; 43:1773–1808. 12401878 10.1194/jlr.r100015-jlr200

